# The relationship among Girdin DNA methylation, its high expression, and immune infiltration in hepatocellular carcinoma: Clues from *in silico* analysis

**DOI:** 10.1042/BSR20204006

**Published:** 2021-03-15

**Authors:** Cheng Zhang, Yang Ke, Xuefen Lei, Xin Liu, Hai Li, Runjiao Shi, Lin Wang

**Affiliations:** 1 Department of Hepatobiliary Surgery, the Second Affiliated Hospital of Kunming Medical University, Kunming 650101, China; 2 Department of Hepatobiliary Surgery, the Sixth People's Hospital of Chengdu, Chengdu 610051, China; 3 Department of Medical Oncology, the Second Affiliated Hospital of Kunming Medical University, Kunming 650101, China; 4 Department of Dermatology, the Second Affiliated Hospital of Chengdu Medical College, Chengdu 610051, China; 5 School of Medicine, Kunming University, Kunming 650214, China

**Keywords:** Girdin, Hepatocellular carcinoma, Immune cells, Methylation, Survival

## Abstract

**Objective:** The aim of the present study was to explore the relationship among Girdin DNA methylation, its high expression, and immune infiltration in human hepatocellular carcinoma (HCC).

**Materials and methods:** The Cancer Genome Atlas (TCGA), Gene Expression Omnibus (GEO), and International Cancer Genome Consortium (ICGC) databases were used to compare Girdin mRNA expression between HCC tissues and normal tissues, and determine the relationship between Girdin expression and HCC prognosis. TCGA database was also used to analyze the expression of Girdin and its methylation status, as well as the relationship between Girdin DNA methylation and HCC prognosis. The Tumor IMmune Estimation Resource (TIMER) database was used to explore the correlation between Girdin expression and HCC immune infiltration.

**Results:** Girdin expression was elevated in HCC tissues compared with that in normal tissues. The degree of methylation at cg03188526, a CpG site in the Girdin gene body, was positively correlated with Girdin mRNA expression, while high Girdin expression and cg03188526 hypermethylation were both correlated with poor HCC prognosis. Additionally, HCC tissue with high Girdin expression exhibited abundant immune infiltration, and the high Girdin expression was associated with a worse prognosis in macrophage-enriched HCC specimens.

**Conclusion:** Our findings indicated that Girdin likely functions as an oncogene in HCC and that hypermethylation at cg03188526 in the Girdin gene body may explain the high Girdin expression levels in HCC tissue. Furthermore, we report for the first time that the adverse effects of high Girdin expression in HCC patients may be partially mediated by tumor macrophage infiltration.

## Introduction

Hepatocellular carcinoma (HCC) is the sixth most commonly diagnosed cancer and the third leading cause of cancer-related deaths worldwide [1]. More than 840,000 new cases of HCC were diagnosed globally in 2018, resulting in approximately 700,000 deaths [2]. Although surgery-dominated, comprehensive individual therapy, including chemotherapy and targeted therapy, has improved the survival of HCC patients to a certain extent, tumor recurrence and resistance to treatment remain key reasons for poor prognosis [3,4]. This highlights the need to identify novel high-precision biomarkers to improve the early diagnosis and treatment of HCC patients.

Girders of actin filaments (Girdin) is a multiple functionally protein (∼216 kDa) consisting of a C-terminal domain with signal-transducing function, a coiled-coil oligomerization domain, and an N-terminal hook domain that can bind to cytoskeletal microtubules [5]. Studies have shown that Girdin possesses diverse biological functions, including as an enhancer of AKT phosphorylation and Gα-binding vesicle-associated protein [5–7]. We have previously demonstrated that Girdin plays a key role in the formation and function of invadopodia in HCC [8]; however, no study to date has explored the relationship among Girdin DNA methylation, its high expression, and immune infiltration in HCC.

Here, The Cancer Genome Atlas (TCGA) database was used to compare Girdin mRNA expression between HCC tissues and normal tissues, to determine the relationship between Girdin DNA methylation and expression, and to analyze the association between Girdin methylation/expression and HCC prognosis. Then, the Gene Expression Omnibus (GEO) and International Cancer Genome Consortium (ICGC) databases, as well as a meta-analysis in combination with TCGA data, were used to verify the correlation between Girdin expression and HCC prognosis. Given the large number of reports on immune cell infiltration in HCC tissues in recent years, the Tumor IMmune Estimation Resource (TIMER) database was also used to explore the correlation between Girdin expression and HCC immune infiltration. Finally, Gene Ontology (GO) enrichment analysis was used to identify the biological processes in which Girdin may participate in HCC.

## Materials and methods

### Data collection

TCGA database (https://gdc-portal.nci.nih.gov/) was searched to obtain the Girdin mRNA and DNA methylation data, as well as the clinical data, of HCC patients [9]. The clinical data included pathological staging, grade, overall survival (OS), progression-free survival (PFS), and family history of cancer. The GEO (https://www.ncbi.nlm.nih.gov/geo/) and ICGC (https://dcc.icgc.org/) databases (a comprehensive gene expression database and a tumor genomics database, respectively), both of which are based on sequencing and array technologies, were also searched to extract the Girdin mRNA expression data as well as the survival data for HCC patients [10,11]. A total of 699 HCC cases (TCGA: 380; GEO-GSE76427: 115; ICGC- LIRI-JP: 203) and 243 normal liver cases (TCGA: 89; GEO-GSE76427: 104; ICGC- LIRI-JP: 50) were included for further analysis [10,11]. The present study conformed with the access rules and release guidelines of TCGA, GEO, and ICGC.

### Meta-analysis

The data from TCGA, GEO, and ICGC databases were meta-analyzed to evaluate the significance of Girdin expression for HCC prognosis. Heterogeneity among the included studies was determined by the *I*^2^-value obtained from the Cochrane *Q* test and the *P*-value obtained from the chi-square test. If there was heterogeneity (*I*^2^ ≥ 50% or *P*<0.05), the results were summarized using a random-effects model. Otherwise, a fixed-effect model was used for analysis. The ´meta’ R package (R version 4.0.0) was used to perform the meta-analysis.

### Immune infiltration analysis

The TIMER database (http://timer.cistrome.org/) was used to comprehensively explore the immunological, clinical, and genomic features of the tumors [12]. Additionally, the TIMER database and its algorithm were employed to analyze the correlation between the abundance of six types of immune cells (CD4+ T cells, CD8+ T cells, B cells, neutrophils, dendritic cells, and macrophages) and Girdin expression in HCC tissues and compare the prognostic data among groups with different levels of Girdin expression and immune cell abundance.

### GO enrichment analysis

GO enrichment analysis was performed using the ´org.Hs.eg.db’ and ´enrichplot’ R packages based on the mRNA sequencing expression profile for HCC in TCGA database [13]. Two-tailed *P*- and *q*-values <0.05 were considered statistically significant.

### Statistical analysis

Statistical analysis was performed using R software (version 4.0.0). The HCC-related mRNA sequencing data extracted from the different databases were all normalized using the ´limma’ package [14]. HCC tissues were divided into high and low Girdin expression groups according to the median value of Girdin mRNA expression from each database. The HCC specimens downloaded from TCGA database were also divided into Girdin hypermethylation and hypomethylation groups based on the median value of the Girdin DNA methylation level. The ´plyr’, ´reshape2’, ´ggpubr’, and ´ggplot2’ R packages were used to map the box and bubble plots. The difference in survival between the high-risk and the low-risk groups was assessed by the Kaplan–Meier method and compared using the log-rank test. A one-way analysis of variance (ANOVA) followed by a Newman–Keuls post-hoc test was used for comparisons among multiple groups. Two-tailed *P*-values <0.05 were considered statistically significant.

## Results

### Girdin expression was up-regulated in HCC and was associated with worse clinical outcome

**Figure 1:**
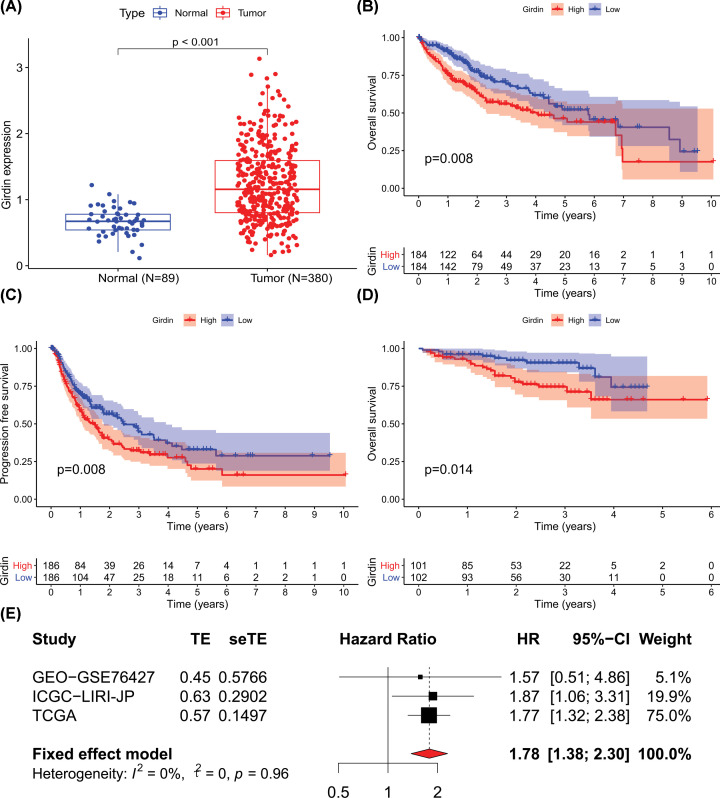
Girdin expression is up-regulated in HCC and is associated with worse clinical outcome (**A**) Girdin expression was significantly higher in HCC tissue specimens than in normal tissues based on The Cancer Genome Atlas (TCGA) data set. Based on TCGA data set, HCC patients with high Girdin expression had poorer (**B**) overall survival (OS) and (**C**) progression-free survival (PFS) than patients with low Girdin expression. (**D**) High Girdin expression was closely related to poorer OS in the ICGC-LIRI-JP data set. (**E**) Meta-analysis with three datasets. High Girdin expression was significantly correlated with poorer OS. TE: estimate of treatment effect; seTE: standard error of treatment estimate.

The analysis of the RNA-sequencing data for 380 HCC and 89 normal tissue specimens from TCGA database showed that Girdin expression was significantly higher in HCC tissue specimens than in normal tissues (Figure 1A). HCC patients in the high Girdin expression group had poorer OS (hazard ratio [HR] = 1.773, 95% confidence interval [CI] = 1.322–2.378, *P*<0.001; Figure 1B) and PFS (HR = 1.716, 95% CI = 1.333–2.209, *P*<0.001; Figure 1C) compared with that of patients in the low Girdin expression group. The ICGC-LIRI-JP data set, including 203 HCC cases and 50 normal liver cases from Japan, showed that high Girdin expression was closely related to poorer OS (HR = 1.873, 95% CI: 1.060–3.308, *P*=0.031; Figure 1D). In contrast, in the GEO-GSE76427 data set (115 HCC cases and 104 normal liver cases from Singapore), no correlation was found between high Girdin expression and OS (HR = 1.572, 95% CI: 0.506–4.882, *P*=0.433; Supplementary Figure S1). Given these contradictory results, we then performed a meta-analysis of the above three data sets to assess the association between Girdin expression and OS and obtain more objective conclusions. As there was no statistical heterogeneity among the three data sets (*P*=0.962, *I*^2^ = 0%), the fixed-effects model was chosen to estimate the combined HR and 95% CI. High Girdin expression was significantly correlated with poorer OS (HR = 1.781, 95% CI: 1.382–2.297, *P*<0.0001; Figure 1E), suggesting that it could be used as a predictor of poor OS.

### The methylation of the cg03188526 site in Girdin DNA was associated with high girdin expression

**Figure 2:**
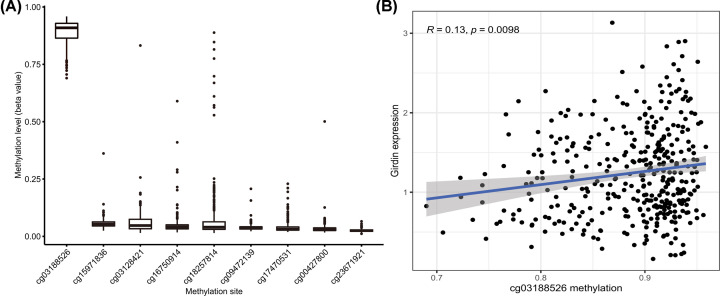
The methylation of the Girdin DNA cg03188526 site was associated with the up-regulation of Girdin expression (**A**) The Girdin DNA cg03188526 site was highly methylated in TCGA-derived HCC tissue specimens. (**B**) The methylation of cg03188526 was correlated with Girdin mRNA expression.

**Figure 3:**
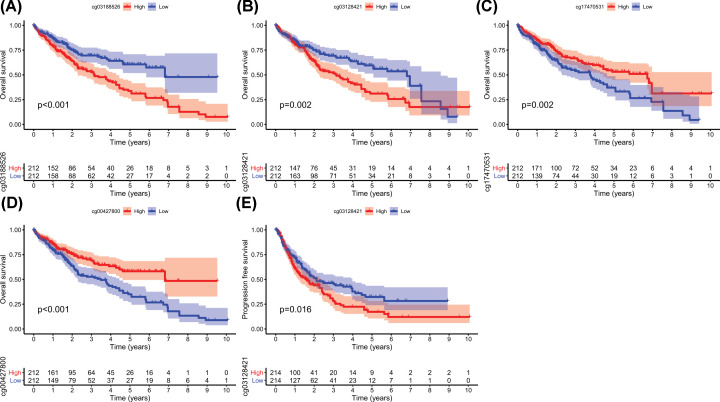
The relationship between the methylation of four Girdin CpG sites and overall survival (OS) or progression-free survival (PFS) in HCC patients Hypermethylation at cg03188526 (**A**) and cg03128421 (**B**) was correlated with poorer OS, while hypermethylation at cg17470531 (**C**) and cg00427800 (**D**) was associated with better OS. Hypermethylation at cg03128421 (**E**) was associated with poorer PFS.

We next sought to identify the cause of the up-regulation of Girdin expression in HCC through analyzing the methylation status of its CpG sites. There were nine CpG sites spanning the whole Girdin gene in TCGA database, including the promoter region [15]. Girdin expression had no correlation with the overall DNA methylation profile or with that of the other eight CpG sites (Supplementary Figure S2A–I); however, the methylation of Girdin cg03188526 was correlated with Girdin mRNA expression (*r* = 0.13, *P*=0.0098; Figure 2B) and cg03188526 was highly methylated when compared with the other eight CpG sites (Figure 2A). We further explored the correlation between the methylation status of the nine CpG sites and OS or PFS in HCC patients. Hypermethylation at cg03188526 (*P*<0.001) and cg03128421 (*P*=0.002) were correlated with poorer OS, whereas hypermethylation at cg17470531 (*P*=0.002) and cg00427800 (*P*<0.001) was associated with better OS (Figure 3A–D). Meanwhile, cg03128421 hypermethylation was associated with poorer PFS (*P*=0.016, Figure 3E). The remaining sites were not significantly correlated with OS or PFS (Supplementary Figure S3A–M).

Finally, we assessed the correlation between Girdin expression or its DNA methylation and the clinicopathological characteristics of the HCC patients from TCGA database. The results revealed that, in HCC patients, high Girdin expression was positively associated with high histological grade and TNM stage, while the overall Girdin DNA hypermethylation status was negatively associated with high histological grade but positively associated with high TNM stage (Table 1).

**Table 1:** Correlation between Girdin expression/methylation and clinicopathological characteristics of HCC in TCGA database

Clinicopathological characteristics		Girdin mRNA expression		*P* value	Girdin DNA methylation		*P* value
		High (%)	Low (%)		High (%)	Low (%)	
Age	<65	100(67.11)	93(62)	0.4217	100(67.11)	93(62)	0.4217
	≥65	49(32.89)	57(38)		49(32.89)	57(38)	
Gender	Female	52(34.90)	46(30.67)	0.5116	52(34.90)	46(30.67)	0.5116
	Male	97(65.10)	104(69.33)		97(65.10)	104(69.33)	
Histologic Grade	G1-2	78(52.35)	102(68)	0.0081^*^	79(52.67)	101(67.79)	0.0107^*^
	G3-4	71(47.65)	48(32)		71(47.33)	48(32.21)	
TNM Stage	Stage I-II	102(68.46)	119(79.33)	0.0444^*^	101(67.79)	120(80.00)	0.023^*^
	Stage III-IV	47(31.54)	31(20.67)		48(32.21)	30(20.00)	
Family history of cancer	Yes	50(33.56)	49(32.67)	0.9675	55(36.91)	44(29.33)	0.2042
	No	99(66.44)	101(67.33)		94(63.09)	106(70.67)	

* *P*<0.005.

### HCC tissue with high girdin expression exhibited abundant immune infiltration

**Figure 4:**
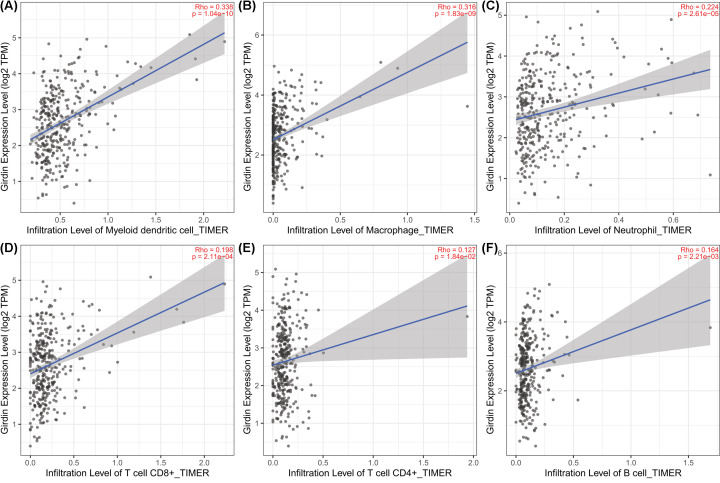
The relationship between Girdin expression and immune cell infiltration in the Tumor IMmune Estimation Resource (TIMER) database Girdin expression was positively correlated with the infiltration of dendritic cells (**A**), macrophages (**B**), neutrophils (**C**), CD8+ T cells (**D**), CD4+ T cells (**E**), and B cells (**F**).

Given the recent success of immune checkpoint inhibitors such as camrelizumab in treating HCC, we comprehensively analyzed the relationship between Girdin expression and immune infiltration in HCC tissue using the TIMER database. In tumors, Girdin expression was positively correlated with immune infiltration, including by dendritic cells (*r* = 0.338, *P*<0.001; Figure 4A), macrophages (*r* = 0.316, *P*<0.001; Figure 4B), neutrophils (*r* = 0.224, *P*<0.001; Figure 4C), CD8+ T cells (*r* = 0.198, *P*<0.001; Figure 4D), CD4+ T cells (*r* = 0.127, *P*<0.001; Figure 4E), and B cells (*r* = 0.164, *P*<0.001; Figure 4F). We further analyzed the relationship between Girdin expression and the expression of markers for the above-mentioned immune cells. Girdin expression was correlated with the CD8+ T cell markers CD8A (*r* = 0.225, *P*<0.001) and CD8B (*r* = 0.145, *P*<0.001); the B-cell markers CD79A (*r* = 0.125, *P*<0.05) and CD79B (*r* = 0.099, *P*<0.05); and the dendritic cell marker CD209 (*r* = 0.307, *P*<0.001) (Supplementary Figure S4A–E).

**Figure 5:**
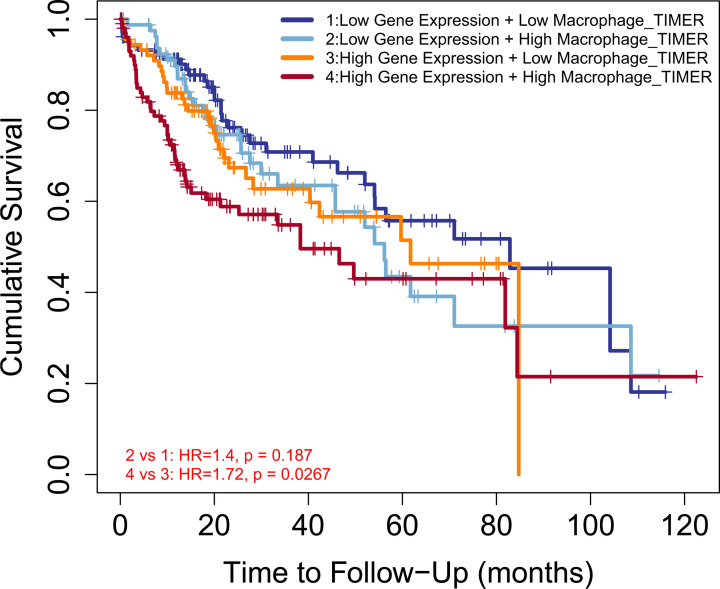
High Girdin expression was associated with worse prognosis in macrophage-enriched HCC specimens

Given that Girdin expression in HCC was closely associated with the infiltration of a variety of immune cells and that elevated Girdin expression was closely related to poor OS, we further clarified whether the adverse effect of high Girdin expression on OS was partially mediated by immune infiltration using the Outcome module in TIMER. As indicated by the Kaplan–Meier survival curve in Figure 5, high Girdin expression was associated with worse prognosis in macrophage-enriched HCC specimens (HR = 1.316, 95% CI: 0.103–1.075, *P*=0.008), but not in those enriched in the other five immune cell types (Supplementary Figure S5A–E), suggesting that the adverse effects of high Girdin expression on the OS of HCC patients may be partially mediated by macrophage infiltration.

### Girdin-related signaling pathway in HCC

**Figure 6:**
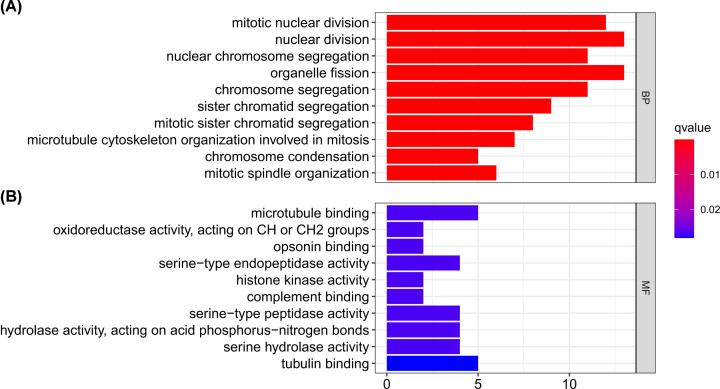
Girdin-related signaling pathways in HCC based on Gene Ontology (GO) enrichment analysis Girdin was found to be mainly involved in the biological processes (BP) of nuclear cell mitosis and cell microtubule skeletal organization (**A**) and the molecular functions (MF) of opsonin binding and complement binding (**B**).

To better clarify the function of Girdin in HCC, as well the underlying mechanisms, GO enrichment analysis was performed using the ‘org.Hs.eg.db’ and ‘enrichplot’ R packages based on the mRNA sequencing expression profile for HCC derived from TCGA database. For biological processes (BP), we found that Girdin was mainly involved in nuclear cell mitosis and cell microtubule skeletal organization (Figure 6A). Interestingly, Girdin was also involved in the molecular functions (MF) of opsonin binding and complement binding, both of which are important in cellular and humoral immunity (Figure 6B).

## Discussion

In the present study, we performed a meta-analysis on 684 HCC samples to systematically analyze the relationship between Girdin expression and the prognosis of HCC patients. We demonstrated that Girdin expression was higher in HCC tissues than in normal tissues and that high Girdin expression was correlated with worse clinical outcome for HCC patients, which was consistent with previously reported results [16–18]. Studies have also indicated that a close relationship exists between dysregulated Girdin expression and poor prognosis in a variety of tumors, including breast cancer and lung cancer [19,20]. The oncogenic effect of Girdin has also been confirmed in several tumors [21,22]. Girdin expression can promote the proliferation, resistance to apoptosis, invasion, and metastasis of tumor cells [17,23,24], all of which are associated with worse clinical outcomes. In terms of the mechanism, Girdin can act as a signal transduction platform to amplify signaling by receptor tyrosine kinases, such as the EGFR and integrins, or by nonreceptor tyrosine kinases, such as Src, which, in turn, stimulates PI3K/AKT signaling [25–27]. We have previously shown that DLG5, Girdin, and TKS5 can interact and regulate DLG5-dependent invadopodia formation in HCC cells [8]. Furthermore, scutellarin suppresses HCC cell invasion by inhibiting Girdin activity [28]. Consistent with these observations, GO enrichment analysis indicated that Girdin was mainly involved in microtubule cytoskeleton organization during cell division and mitosis.

Interestingly, we found that the degree of methylation at cg03188526 was positively correlated with Girdin mRNA expression, and that Girdin DNA cg03188526 was highly methylated in HCC tissues. DNA methylation represents an important means of modifying gene expression and is closely related to tumor occurrence and development [29]. We noticed that the cg03188526 site is located in the gene body of Girdin. Unlike the hypermethylation of gene promoter regions, which is often closely associated with gene silencing [30], gene body methylation is not thought to be related to gene silencing, but may instead promote gene expression and alternative splicing [31,32]. Consequently, we reasoned that the cg03188526 methylation level would affect Girdin expression or its alternative splicing. In keeping with this, we found that the cg03188526 methylation level in HCC was also correlated with the prognosis of HCC patients.

Additionally, the TIMER database was used to reveal for the first time that Girdin expression in HCC is correlated with the infiltration of a variety of immune cell types, and that the adverse effects of high Girdin expression on the OS of HCC patients may be partially mediated by macrophage infiltration of the tumor microenvironment. With the progress of immunotherapy, research has increasingly focused on the putative correlations between tumors and immunity [33]. The level of tumor infiltration by immune cells is correlated with tumor growth, progression, and patient outcome [34], and studies have also reported that a close relationship exists between immune infiltration and HCC occurrence and development [35,36]. However, no study has analyzed the association between Girdin expression and immune cell infiltration. Here, we showed that the expression of Girdin in HCC was correlated with moderate to low levels of infiltration of dendritic cells, macrophages, neutrophils, CD8+ T cells, CD4+ T cells, and B cells. Importantly, we found that high Girdin expression was correlated with worse prognosis in macrophage-enriched HCC specimens, but not in those enriched with the other five immune cell types. Recent studies have demonstrated that there is a positive correlation between the expression of programmed cell death 1 ligand 1 (PD-L1) and tumor cell-intrinsic osteopontin (OPN), key drivers of macrophage infiltration and immune escape in HCC, and that both were associated with HCC development [37]. GO enrichment analysis indicated that Girdin is involved in opsonin binding and complement binding in HCC. Recent studies have shown that high levels of complement activation and the infiltration of tumor-associated macrophages can maintain chronic inflammation, promote an immunosuppressive microenvironment, induce angiogenesis, and increase the motility and metastatic potential of cancer cells [38,39]. The present study is the first to indicate that the adverse effects of high Girdin expression on the OS of HCC patients may be partially mediated by macrophage infiltration.

The present study had several limitations. First, although our *in silico* results suggested that methylation at a specific site in the Girdin gene body and Girdin expression are correlated with poor prognosis in HCC, cell- and animal-based experiments are needed to validate these findings. Second, we could not verify that a correlation exists between Girdin DNA methylation and PFS in the GEO-GSE76427 and ICGC-LIRI-JP data sets because they do not include information for PFS and Girdin DNA methylation. Third, given that this was an exploratory study and a significance level of 5% was selected, no adjustment for multiple testing was performed [40]. However, it is worth noting that, irrespective of the study type, not adjusting for multiple comparisons may result in an inflation of the false-positive rate [40].

## Conclusion

In the present study, we found that high Girdin expression and cg03188526 hypermethylation were correlated with poor prognosis for HCC patients, while hypermethylation at the cg03188526 site in the Girdin gene body may explain, at least in part, its high expression in HCC. Finally, the adverse effect of high Girdin expression on the OS of HCC patients may be partially mediated by macrophage infiltration of the tumor microenvironment.

## Supplementary Material

Supplementary Figures S1-S5

## Data Availability

The data sets used and/or analyzed during the current study are available from the corresponding author on reasonable request.

## Abbreviations

GEO: Gene Expression Omnibus
HCC: hepatocellular carcinoma
ICGC: International Cancer Genome Consortium
OPN: osteopontin
OS: overall survival
PFS: progression-free survival
PD-L1: programmed cell death 1 ligand 1
TCGA: The Cancer Genome Atlas
TIMER: Tumor IMmune Estimation Resource
