# Supplementary material for: The relationship among Girdin DNA methylation, its high expression, and immune infiltration in hepatocellular carcinoma: Clues from in silico analysis
Source: Biosci Rep. 2021 Mar 15;41(3):BSR20204006. doi: 10.1042/BSR20204006 (PMC7960887; doi:10.1042/BSR20204006)
Supplement: Supplementary Figures S1-S5 [file BSR-2020-4006_supp.pdf]

Supplementary Material

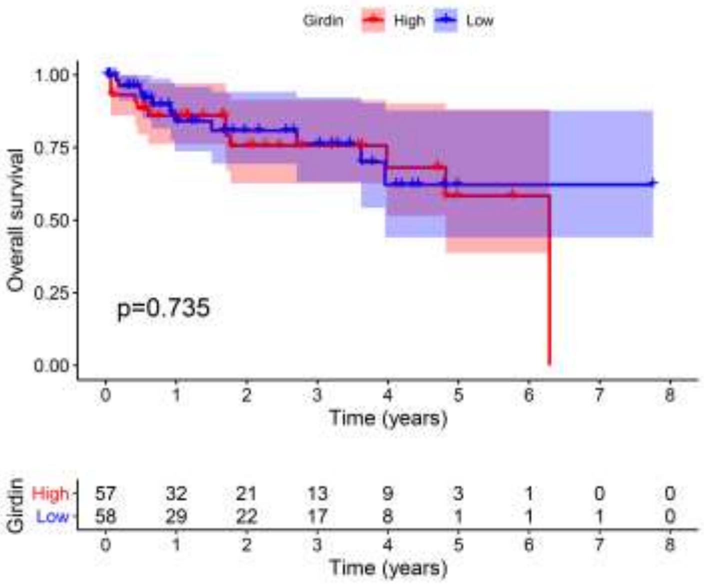

Figure S1 High expression of Girdin was not correlated with OS in GEO-GSE76427 dataset.

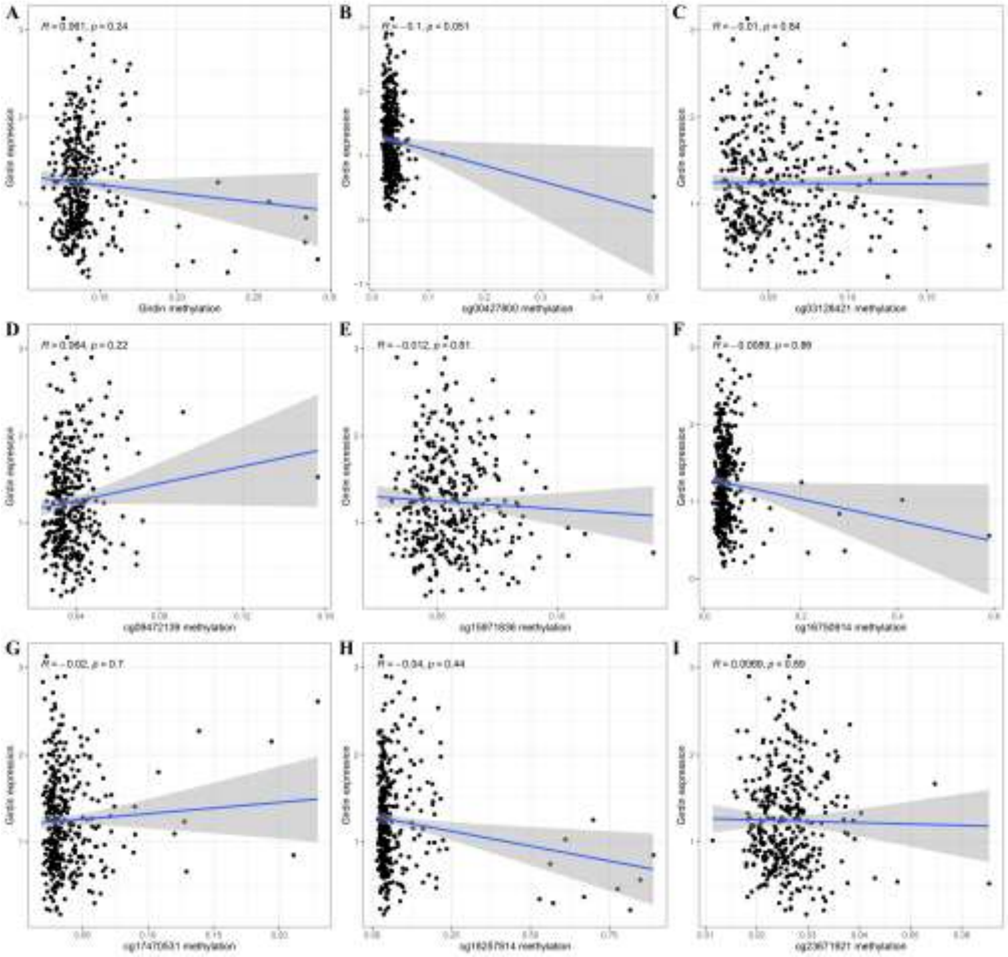

Figure S2 Neither the overall methylation status of Girdin DNA (A) nor 8 CpG sites of Girdin DNA (B-I) were correlated with the Girdin expression.

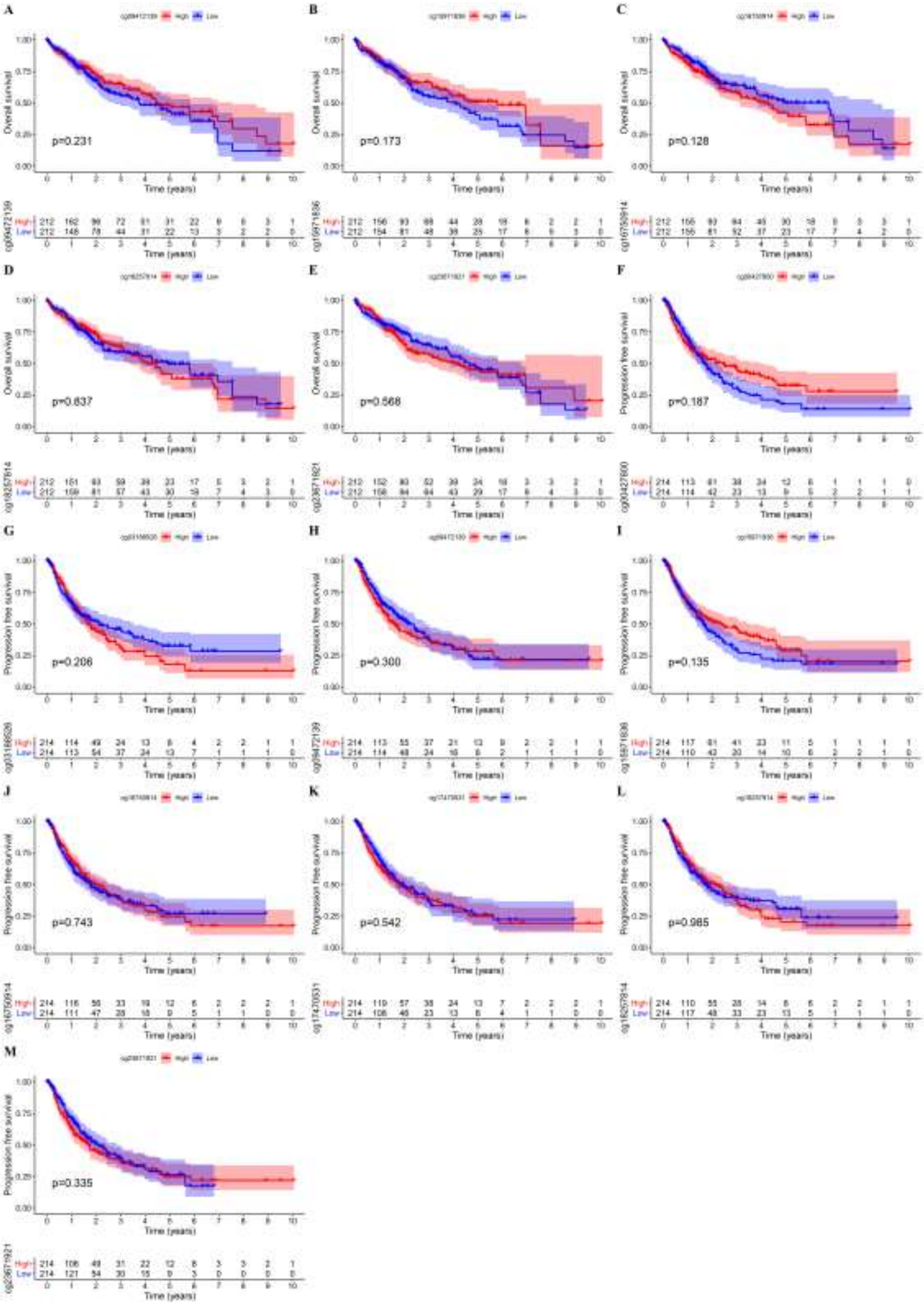

Figure S3 Some hypermethylation at Girdin DNA CpG sites were not associated with PFS nor OS in HCC patients. Methylation at (A) cg09472139, (B) cg15971836, (C) cg16750914, (D) cg18257814, and (E) cg23671921 were correlated with OS in HCC patients. And methylation at (F) cg00427800, (G) cg03188526, (H) cg09472139, (I) cg15971836, (J) cg16750914, (K) cg17470531, (L) cg18257814, (M) cg23671921were not associated with PFS.

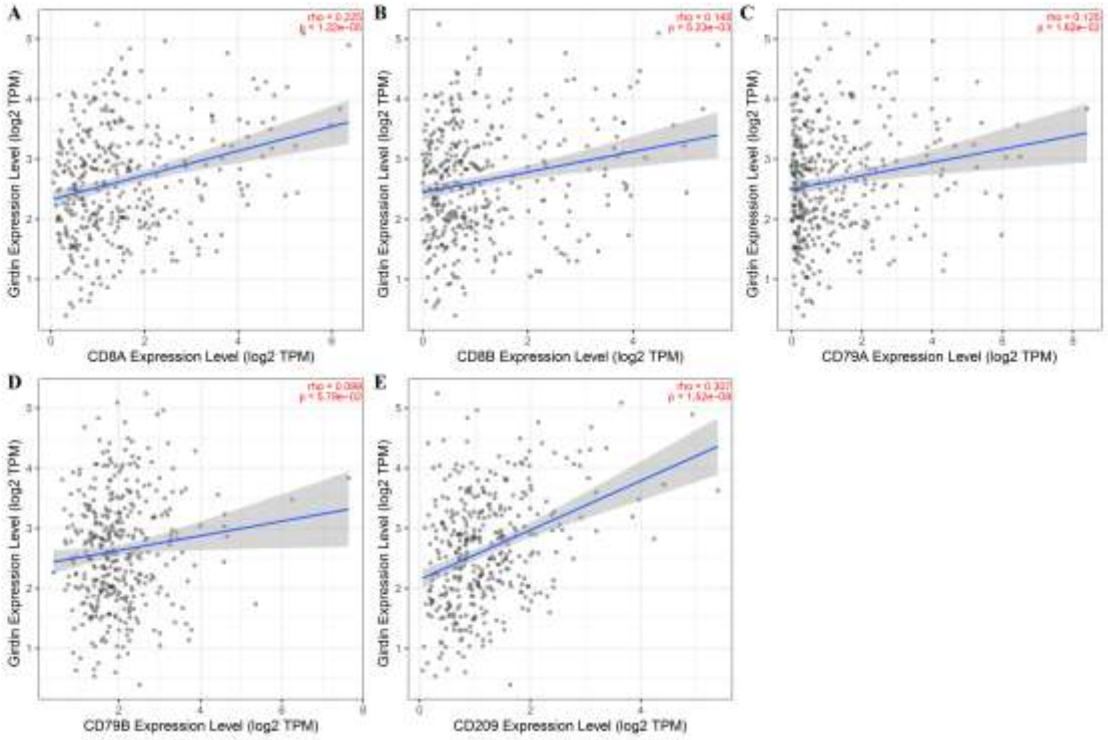

Figure S4 Girdin expression was correlated with immune markers (A) CD8A and (B) CD8B of CD8+ T cell, immune markers (C) CD79A and (D) CD79B of B cell, and immune marker (E) CD209 of dendritic cell in TIMER database.

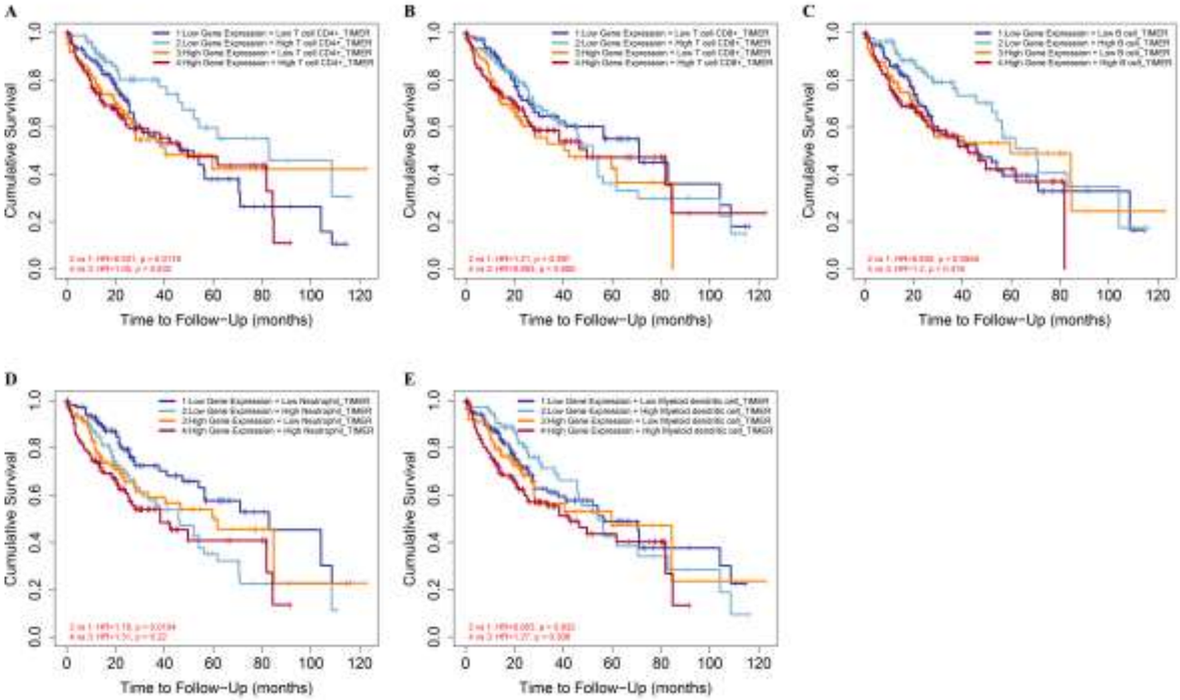

Figure S5 There was no significant correlation between high expression of Girdin and worse prognosis in the HCC specimens with (A) $CD4^+$ T Cells, (B) $CD8^+$ T Cells, (C) B cells, (D) neutrophils, and (E) dendritic cells enriching.
